# Integrating Radiomics Signature into Clinical Pathway for Patients with Progressive Pulmonary Fibrosis

**DOI:** 10.3390/diagnostics15030278

**Published:** 2025-01-24

**Authors:** Giacomo Sica, Vito D’Agnano, Simon Townend Bate, Federica Romano, Vittorio Viglione, Linda Franzese, Mariano Scaglione, Stefania Tamburrini, Alfonso Reginelli, Fabio Perrotta

**Affiliations:** 1 Radiology Unit, Monaldi Hospital, A.O. dei Colli, 80131 Naples, Italy; federica.romano@ospedalideicolli.it; 2 Department of Translational Medical Sciences, University of Campania “L. Vanvitelli”, 80131 Naples, Italy; franzeselinda@gmail.com (L.F.); fabio.perrotta@unicampania.it (F.P.); 3 Lungs for Living Research Centre, UCL Respiratory, University College London, London WC1E 6BT, UK; simon.bate@ucl.ac.uk; 4 Department of Precision Medicine, University of Campania “L. Vanvitelli”, 80138 Naples, Italy; vittorioviglione@gmail.com (V.V.); alfonso.reginelli@unicampania.it (A.R.); 5 U.O.C. Clinica Pneumologica L. Vanvitelli, Monaldi Hospital, A.O. dei Colli, 80131 Naples, Italy; 6 Radiology Department of Surgery, Medicine and Pharmacy, University of Sassari, 07100 Sassari, Italy; mscaglione@uniss.it; 7 Department of Radiology, Ospedale del Mare, ASL NA1 Centro, 80147 Naples, Italy; tamburrinistefania@gmail.com

**Keywords:** interstitial lung diseases, ILD, radiomics, artificial intelligence, deep learning, progressive pulmonary fibrosis

## Abstract

Interstitial lung diseases (ILDs) are a heterogeneous group of pulmonary disorders characterised by variable degrees of inflammation, interstitial thickening, and fibrosis leading to distortion of the pulmonary architecture and gas exchange impairment. There are approximately 200 different entities in this category. ILDs are commonly classified based on several criteria, including causes, clinical features, and radiological patterns. Chest HRCT is the gold standard for the recognition of lung alteration patterns underlying interstitial lung diseases (ILDs), diagnosing specific patterns, and evaluating radiologic progression. Methods based on artificial intelligence (AI) may be used in computational medicine, especially in image-based specialties such as radiology. The evolving field of radiomics offers a unique and non-invasive approach to extracting quantitative information from medical images, particularly high-resolution computed tomography (HRCT) scans. This comprehensive review explores the burgeoning role of radiomics in unravelling the intricacies of interstitial lung disease. It focuses on its potential applications in diagnosis, prognostication, and treatment response evaluation.

## 1. Introduction

Interstitial lung disease (ILD) is a generic term referring to a heterogeneous group of pulmonary disorders characterised by variable degrees of inflammation and interstitial thickening, leading to distortion of the pulmonary architecture and fibrosis [1,2,3]. Idiopathic pulmonary fibrosis (IPF) represents the archetype of progressive fibrotic lung diseases. However, evidence shows that clinical courses of patients with ILDs other than IPF, such as connective tissue disease (CTD)-ILDs, fibrotic hypersensitivity pneumonitis (F-HP), idiopathic nonspecific interstitial pneumonia (iNSIP), unclassifiable ILD (u-ILD), chronic sarcoidosis, may follow that of untreated IPF. These subsets of patients may benefit from antifibrotic treatment [4]. In this respect, an accurate and prompt diagnosis is of paramount importance to enable risk stratification and treatment initiation [5]. A multidisciplinary approach, involving a complex integration of clinical, functional, radiological, and, when available, pathological data, is now considered the gold standard for ILD classification beyond IPF [6,7]. High-resolution computed tomography (HRCT) represents a rapid, repeatable, and safe technique that plays a pivotal role in the recognition of lung alteration patterns, evaluating radiological progression, and quantifying comorbid conditions of the thorax. In addition, it also plays a prognostic role in fibrotic lung diseases [8,9,10]. Coupled with clinical and functional assessments, the visual score of HRCT scans has served as the underpinning pillar for evaluating patients with ILDs for more than a decade since their introduction [11]. However, visual assessment of ILDs through HRCT, although integrated with pathophysiological data, can be prone to substantial inter-reader variability, even between skilled radiologists [7,12,13]. In addition, it is worth considering that radiologists and multidisciplinary teams do not always have the necessary expertise to evaluate HRCT in ILD patients with potential diagnostic delays up to 2 years [14,15]. Conversely, employing computer-based methodologies to analyse HRCT images in the worldwide valid DICOM format, quantitative CT analysis (QCTA) offers an alternative assessment approach that remains both objective and replicable [11,16,17]. Recent advancements in computing power have facilitated rapid progress in image analysis, including endeavours to leverage machine learning for the development of more precise and specific digital biomarkers.

This review provides an overview of technological advancements in QCTA supporting the diagnosis and monitoring of ILDs. In addition, we describe how machine learning (ML)-based approaches can be applied to identify new predictive biomarkers of response to antifibrotic treatment.

## 2. Methods

A systematic literature search was conducted by Medline, Scopus, and the Cochrane Database, including articles published in the last 10 years (from 2015 to 2025). Only articles written in English were included. The following MESH terms were used: artificial intelligence, machine learning, deep learning, neural networks, AI, natural language processing and interstitial lung diseases, pulmonary fibrosis, idiopathic pulmonary fibrosis, ILDs, interstitial pneumonia, connective tissue disease-associated ILD. Therefore, we report a narrative review of the abovementioned research.

## 3. Artificial Intelligence for Detecting Fibrotic ILD Patterns in Computed Tomography

### 3.1. First Order Statistics

Several computed-based quantification techniques have been proposed as alternatives to visual scoring for enhancing objectivity, sensitivity, and reproducibility in change detection in medical images of ILD patients.

QCTA is defined as the conversion of digital medical images into mineable high-dimensional quantitative data [18]. Based on pulmonary tissue properties to variably attenuate X-rays, lung densitometry represents a straightforward QCTA technique that assesses the density of individual pixels or voxels as well as manually, automatically, or semi-automatically selected regions of interest (ROIs) for the entire lung. This digital sampling successively allows the computational production of quantitative metrics, such as attenuation (HU) threshold-based measurements as well as first-order histogram-based statistics.

First-order CT pixel histogram-based statistics include mean, median, and metrics based on lung attenuation, such as variance, kurtosis, skewness, and entropy. Kurtosis describes the sharpness of the histogram peak, which negatively correlates with the thickness of the two tails of the histogram. Skewness refers to the extent of histogram asymmetry. Although qualitative visual assessment is required before quantitative analysis, the advantages of lung densitometry have been recognised in terms of its greater sensitivity of pathological variations and evaluation of all contiguous sections. In this regard, modern quantitative tools can segment the lungs almost flawlessly without additional assistance or correction from the operator, enhancing the objectivity and reproducibility of quantitative analyses [19]. Mean, median lung attenuation, skewness, and kurtosis are surrogates of pulmonary diffuse diseases severity and extent, such as chronic obstructive pulmonary diseases, and pulmonary fibrosis [20]. Lung fibrosis or inflammation causes an increase in soft tissue of the lung, which will increase the mean lung attenuation and decrease kurtosis. Whilst a low kurtosis is associated with reticulation and honeycomb to a large extent, a high kurtosis reflects less reticulation and correlates with normal spirometry. Kurtosis and skewness of a histogram are correlated with changes in FVC and overall survival in patients with ILDs [21,22]. As demonstrated by Torrisi et al., kurtosis skewness and Mean Lung Density (MLD) were good predictors of mortality in a cohort of 42 patients with IPF (HR 0.44, 0.74, 1.01, *p* = 0.03, *p* = 0.01, *p* = 0.02, respectively) [23]. In addition, subclinical parenchymal lung diseases defined as high attenuation areas (HAAs) with a CT attenuation value ranging between −600 and −250 HU in a lung field have been associated with a shorter transplant free survival [17,24].

Combining mean lung attenuation (MLA), skewness, and kurtosis from low-dose thin-section volumetric lung CT scans in a cohort of patients affected systemic sclerosis-associated interstitial lung disease (SSc-ILD). Bocchino et al. proposed a single composite densitometric index named the Computerized Integrated Index (CII). The authors demonstrated that the proposed CII identified the presence of ILD with diagnostic accuracy, sensitivity, and specificity of 77%, 81%, and 66%, respectively. This suggests that the CII may be more sensitive than visual scoring, potentially aiding in early detection of ILD in SSc patients [25].

### 3.2. Higher-Order Statistic and Machine Learning Approaches

One of the recognised drawbacks of first-order statistics is that they do not consider the spatial relationship between voxels; consequently, results may be confusing, especially when lung fibrosis and obstructive diseases coexist. In addition, similar results may be obtained by density histograms of different ILDs, leading to limitations in terms of differential diagnosis [11].

In this respect, higher-order statistics can examine location as well as the spatial relationship between pixels or voxels. In addition, image filtration can be performed to reduce, for example, image noise or enhance edges. It can also increase the number and variety of image features. Consequently, a feature vector generated by linking together these multiple features can be used as a signature for other given images.

**Figure 1:**
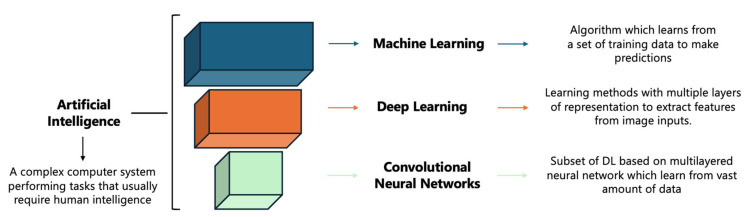
Matryoshka structure of artificial intelligence family. DL: Deep Learning.

The term “artificial intelligence” (AI) refers to a complex computer system performing tasks that usually require human intelligence (Figure 1). AI is characterised by a multi-level labyrinth of different hierarchical stages of neural networks. These range from the most superficial step, represented by machine learning—an algorithm that extracts salient features from a training dataset rather than being programmed with specific instructions—to the deepest and least explored stage, represented by convolutional neural networks (CNN) [16,23,24]. Broadly, machine learning can be grouped into two categories: supervised and unsupervised. Whilst in supervised learning (such as a support vector machine), prelabelled data are provided to the machine to develop a method for predicting labels. In unsupervised learning, the machine independently groups the data, without prelabelled validation. Currently, machine learning quantitative tools rely on supervised as well as a combination of both unsupervised and supervised learning. In recent years, the growing use of a higher-order statistical model of Texture Analysis (TA) has gained popularity as a promising AI method for ILD diagnostics, as it provides a quantitative assessment of lung tissue features. Quantitative information is extracted from the spatial distribution of pixel intensities in radiological images by TA using computational algorithms.

Computer-Aided Lung Informatics for Pathology Evaluation and Rating (CALIPER) is a software application developed by Biomedical Imaging Resource Laboratory at the Mayo Clinic Rochester (Rochester, MN, USA) known for its effectiveness and widespread use in texture analysis by combining a supervised and unsupervised learning approach [26]. In CALIPER, randomly selected volumes of interest (VOIs) from lung parenchyma are initially categorised into one among five different patterns (normal, emphysema, ground-glass, reticular, and honeycomb) by expert radiologists. Histogram-based techniques are then successively tested to evaluate their pattern discrimination capabilities. Next, each voxel of a given CT image is classified according to the histogram features of the surrounding VOIs. In a cohort of 55 patients with IPF, Maldonado et al. demonstrated that total reticular changes that occur within a short period (3–15 months), as well as the percentage and total ILD changes, were predictive of survival when evaluated through CALIPER [27]. Conversely, changes in individual ILD parenchymal findings were not associated with survival when analysed by radiologists, despite their expertise. However, the overall global change was predictive of survival even when analysed by radiologists. Mild to moderate correlations have been found between radiologists and CALIPER regarding ground glass and reticular findings [27]. In addition, computer QCTA may reveal features misidentified by visual analysis. Jacob et al. have demonstrated that quantitation of vessel-related structures (VRSs), a collective term comprising pulmonary vessels (arteries and veins) and associated structures (perivascular fibrosis), can significantly predict survival and likelihood of FVC decline in patients with less extensive disease. Importantly, authors reported that VRS scores successfully identify patients with IPF who will reach drug trial endpoints and respond to antifibrotic medication, suggesting QCTA’s strategical role in reducing the prohibitive costs of current IPF trials [28].

Imbio platform is another artificial intelligence software based on the CALIPER algorithm. The fully automated LTA tool uses advanced computer vision to turn a standard CT scan into a detailed map. It also quantifies lung textures key to identifying ILD and other fibrotic conditions. LTA algorithm’s volumetric scoring system classified patterns in opacities, reticular patterns, honeycombing, hyperlucent, normal lung, and VRS [29].

In a retrospective cohort of 35 consecutive patients with SSc-ILD during treatment follow-up, variation in total lung volume at QA performed using Imbio LTA accurately predicted changes in the composite functional respiratory endpoint with FVC% and DLco% (AUC = 0.74; 95%CI: 0.54 to 0.93; *p* = 0.03) [30,31].

## 4. Deep Learning Algorithms and Convolutional Neural Networks for Quantitative Texture Analysis in Fibrotic ILDs

Recognizing early signs of fibrosis and distinguishing ILDs into fibrotic and non-fibrotic are crucial to prompt treatment initiation. Deep learning, a form of machine learning based on CNNs, represents a key support in computer-aided detection/diagnosis (CAD) process [32]. Specifically, deep learning models, consisting of highly interconnected hierarchical layers of non-linear processing units, can automatically analyse and interpret medical images with progressive optimization of those layers responsible for feature extraction, selection, and classification. In turn, these abilities have led to significant improvements in several processing tasks, such as automatic segmentation, registration, and pattern classification of medical images [11,30]. Large datasets of CT images can be used to train DL models to automatically and reproducibly detect and classify various ILD patterns and abnormalities [33]. For radiologists and clinicians with little expertise in thoracic imaging, this detection is of paramount importance. Interestingly, Nikishiori et al. demonstrated that deep-learning algorithms significantly outperform both radiologists and pneumologists in detecting chronic fibrosing ILDs in terms of sensitivity and specificity using chest radiograph images, suggesting DL models’ key role in early ILDs diagnosis [33].

Data-driven texture analysis (DTA) is a DL-based texture analysis tool for diffuse parenchymal lung diseases that uses a combination of supervised and unsupervised machine learning. Briefly, after an unsupervised production of a dictionary of features distinguishing fibrosis from non-fibrotic regions in CT scans of patients with IPF and non-smokers control, quantitative features obtained by radiologists labelled ROIs from the same cohort are matched with the unsupervised dictionary. A support vector machine (SVM) classifier is then obtained to classify sections as either normal lung or fibrosis. The DTA fibrosis score is computed as a percentage of the total number of window regions classified as fibrosis [34]. Humpries et al. demonstrated that baseline DTA score was significantly associated with the annual rate of decline in both FVC and DLCO. In multivariable Cox proportional hazard models, a greater extent of lung fibrosis was significantly associated with worst progression-free survival (HR 1.14, *p* <  0.0001) and poorer transplant-free survival (hazard ratio [HR] 1.20, *p* <  0.0001) [35]. Further examples of deep learning models are, among others, the Systematic Objective Fibrotic Imaging Analysis Algorithm (SOFIA), developed and validated in UIP-like features identification, the Quantitative Lung Fibrosis (QLF) score, which estimates the extent of reticular patterns in computed tomography (CT), and QUIBIM, an AI-based deep learning algorithm that provides promising indicators to lung cancer risk in a cohort of IPF patients [36].

## 5. Novel Perspective on AI Prognostic Quantification and Predicting Response to Treatment

**Figure 2:**
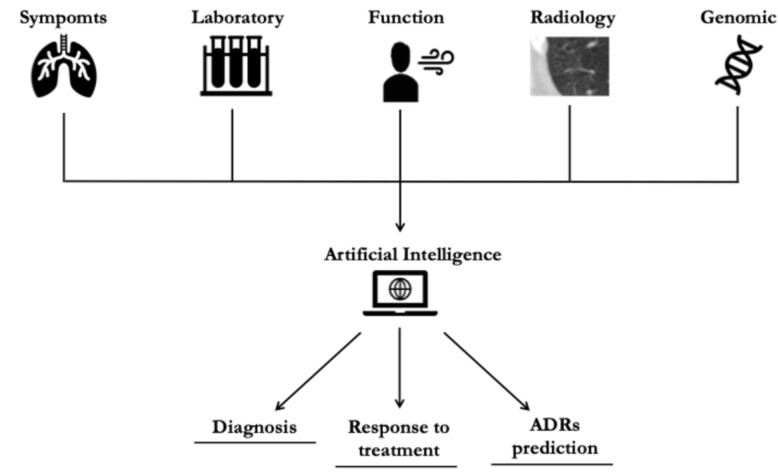
Role of AI-based analysis in diagnosis, response to treatment, and adverse drug reaction. ADR: adverse drug reactions.

Since the approval of antifibrotic treatment for progressive fibrotic ILDs, identifying biomarkers capable of predicting fibrotic disease progression has challenged scientific societies. Currently, the definition of progressive pulmonary fibrosis (PPF) is based on longitudinal evidence of worsening respiratory symptoms, a decline in FVC or DLCO, and radiological disease progression [37]. Reliable identification of progressive fibrotic lung disease using both baseline functional and imaging clinical data may help healthcare providers provide early treatment initiation, which would positively impact patients’ outcomes [37,38]. Therefore, the practicality and reliability of the morphological assessment through HRCT examination have significant potential for enhancing patient management, predicting treatment response, and tailoring treatment strategies (Figure 2).

The growing use of quantitative software, which incorporates artificial intelligence (AI), offers a more objective approach to analysing ILDs due to its ability to expand beyond the limits of human radiological vision [11]. Integrating imaging data with clinical, demographic, functional, and other -omic features, radiomics may also play a key role in identifying patients at higher risk of disease progression, facilitating timely interventions and personalised care. In addition, radiomics may also help healthcare providers identify new, sensitive, reproducible, and objective biomarkers for tracking disease progression and response to therapy.

Trends in forced vital capacity (FVC) or carbon monoxide diffusing capacity (DLCO) are routinely used as a primary endpoint in ILD clinical trials but are not a proven surrogate for mortality [39,40]. AI-based QCTA can be reproducibly performed. These advanced computational tools offer an objective analysis of lung abnormalities and their extension, measure baseline disease severity and progression, and may support clinicians in assessing antifibrotic treatment response, overcoming the limitations of subjective and visual evaluations. Several computer-assisted diagnosis algorithms for HRCT pattern classification and ILD prognostication have been proposed. Yet, consensus still lacks regarding the QCT analysis method of choice.

Kim et al. compared two well-established QCT approaches, the CT histogram metric and the QLF score, based on their ability to assess not only baseline severity but also changes over a short period in a cohort of 57 patients with IPF. The authors demonstrated that both kurtosis and the QLF score are associated with pulmonary function at baseline and overall survival. However, QLF was the only approach capable of detecting temporal changes in reticulation extent [41].

The adaptive multiple features method (AMFM) represents an automated HRCT analysis program developed by the University of Iowa’s Department of Radiology (Iowa City, IA, USA) Pulmonary Analysis Software Suite 9.0. AMFM can be trained to recognise and quantify the volume occupied by a variety of radiologic patterns, such as ground-glass (GGO), reticular GGO (GGR), honeycombing, and emphysema. Interestingly, greater baseline volume occupied by GGR in the AMFM assessment was associated with increased hazard of disease progression (hazard ratio [HR] per 10% AMFM GGR increase = 1.37, 95% CI = 1.07–1.75, *p* = 0.01), even after adjusting for relevant covariates (HR per 10% AMFM GGR increase = 1.36, 95% CI = 1.01–1.84, *p* = 0.04) [42].

Coupled with functional changes in FVC and DLCO, a longitudinal classifier-driven HRCT metric may provide a more robust measure of disease progression.

Deep learning has been increasingly applied to both the diagnostic process and the management of patients with ILD. It can autonomously identify different patterns in high-dimensional data, mapping them to end-points such as disease progression. This ability undoubtedly represents the key advantage of deep learning: the computer extracts the most predictive features from images in an autonomous fashion. Therefore, it is possible to identify novel HRCT biomarkers, including those not readily identified visually. Several studies have investigated the utilization of CNNs and AI to predict and diagnose interstitial lung diseases [8,43].

For example, a study has investigated the prognostic utility of the deep learning algorithm SOFIA, a deep convolutional neural network loosely based on the Inception-ResNet-v2 architecture, which has been trained and validated for the identification of UIP-like features. In their study, Walsh et al. utilised SOFIA to provide a “UIP probability” score, representing the probability of each of the UIP diagnosis categories (definite, probable, indeterminate, alternative) for each given slide. In a cohort of 504 patients with fibrotic lung disease, only SOFIA UIP probabilities were significantly predictive of transplant-free survival, also when adjusted for total ILD extent (HR, 1.06; *p* < 0.0001; 95% CI, 1.04–1.08) [8]. Then, SOFIA and radiologist UIP probabilities were converted into PIOPED-based UIP probability categories, demonstrating that only the SOFIA-PIOPED UIP probability categories were predictive of transplant-free survival, even after multivariable analysis adjusting for age, sex, and total ILD extent [8]. Interestingly, it has been reported that an automated simple automatic opacity-based fibrotic lung (OFL) measurement through a binary classification of tissue into either normal or opacified lung displayed similar results and prognostic values compared to a more complex multi-texture-based analysis [44].

The application of artificial intelligence is not limited to quantitative analysis of fibrotic ILDs. It also may help clinicians develop novel algorithms capable of analysing not only radiological data, but also complex biological and molecular data, offering new perspectives on identifying novel biomarkers and therapeutic targets. Recently, Zhou et al. merged single-cell sequencing technologies with AI, revealing two key genes—PHACTR1 and BLVRB—in pulmonary fibrosis pathogenesis. Based on data from diagnostic ROC analysis, the area under the curve (AUC) for both PHACTR1 and BLVRB was 0.986, indicating excellent diagnostic efficacy. Interestingly, the authors demonstrated that both PHACTR1 and BLVRB genes positively correlate with several types of immune cells infiltration, including Type 17 T helper cells, macrophages, monocytes, MDSC, and neutrophils. Conversely, animal models of IPF, PHACTR1 knockout mice exhibited a significant reduction in lung tissue inflammation and collage deposition compared with the control group [45]. Similarly, machine learning LASSO regression analysis and RT-qPCR has demonstrated statistically significant NSUN6 expression in RA-ILD [46]. Using PandaOmics, a commercially available target-discovery platform using multiple AI engines, Ren et al. identified a novel inhibitory molecule of TRAF2 and NCK Interacting Kinase (TNIK)—a member of the serine–threonine kinase STE20 family—named INS018_055, which has been shown to ameliorate TGF-β-induced EMT and FMT signalling in vitro. It also attenuates murine bleomycin-induced lung fibrosis alone and in combination with pirfenidone [47]. In addition, two phase I studies demonstrated the safety and tolerability of INS018_055 in healthy volunteers with good oral bioavailability and dose-proportional PK [47].

## 6. Radiomics in ILDs: Current Evidence and Future Perspectives

Radiomics holds great promise in enhancing our understanding of ILDs and its application to the landscape of ILDs has grown over the last decade. Providing valuable insights into disease pathogenesis, progression, and treatment response, radiomics integrates different shape-, intensity-, texture-, and wavelet-based features with clinical, laboratory, and lung function data. Subsequently, these hand- or automatic-crafted features may be useful for training machine learning classifiers and developing more accurate disease severity predictions [48]. To date, several studies examining the diagnostic and therapeutic implications of radiomics in fibrotic ILDs (Table 1) and CTD-related (Table 2) progressive pulmonary fibrosis have been published. With respect to IPF, the prototype of progressive pulmonary fibrosis, radiomics may outperform visual assessments [49,50]. More specifically, Rafaee et al. achieved a sensitivity of 98% and a specificity of 98% to identify an ILD, using all hand-crafted radiomic features, highlighting the potential role of radiomics in reducing the amount of time needed by clinicians for image assessment [49]. Haga et al. extracted radiomics using artificial intelligence-based software and PyRadiomics in a cohort of ILD patients undergoing surgical lung biopsy (SLB). In this study, radiomics predicted inflammatory cellular infiltration within the fibrotic area, supporting its role in the treatment planning of patients with ILDs [51]. Similarly, radiomics has been shown to support clinicians in disease severity stratification and treatment planning as well as patients with CTD-ILDs. Indeed, radiomics is not only useful for predicting the GAP stage in this subset of patients, outperforming clinically-based models for predicting mortality [52,53], but it also reduces radiation exposure [54]. Using in-house developed radiomics software called Z-Rad, Joye et al. demonstrated that radiomics from slice-reduced CT (srCT) is an effective and preferable alternative to full-chest CT (fcCT) for both diagnosing and staging ILD in patients with SSc-ILD [54], decreasing the amount of radiation needed. Capable of inducing fibroblast migration and inhibiting cell–cell adhesion, Krebs von den Lungen-6 (KL-6), a glycoprotein mainly expressed by damaged alveolar type II cells, has emerged as a promising biomarker for interstitial lung fibrosis [55,56]. A nomogram model combining radiomic features with serum KL-6 predicted RA-ILD low-risk patients, according to the GAP stage, with an AUC of 0.948 and 0.923 in the training and validation cohorts, respectively [57]. Moreover, radiomics could also predict mortality in this subset of patients, as demonstrated in a proof-of-concept study by Venerino et al. (median grey level intensity within the whole lung segmentation [high-resolution (HR) 9.35, 95% CI 1.56–55.86] [58].

Anti-melanoma differentiation-associated gene 5-positive dermatomyositis (MDA5^+^ DM) represents a rare autoimmune disease predominantly reported in East Asia and is characterised by impressively high mortality due to rapid-progressive interstitial lung disease (RP-ILD). The role of radiomics in patients with MDA5^+^ DM-ILD has been investigated. In a multi-centre retrospective study, He et al. generated four risk scores based on radiomic features extracted from four areas of HRCT. They were subsequently integrated with selected clinic–radiologic variables, including consolidation score, LDH, and infection to build a nomogram. The survival analysis showed that patients with higher nomogram scores had worse survival outcomes than those with lower scores in two different institutions [HR = 4.117 (95% CI = 2.195–7.722; *p* < 0.001) and HR = 7.515 (95% CI = 1.297–43.540, *p* < 0.001), respectively] [59].

Glucocorticoids represent the cornerstone of treatment for patients with Systemic Autoimmune Rheumatic Diseases other than SSc-ILD [60]. Nonetheless, a subset of patients, reaching 30% in rheumatoid arthritis, show poor clinical response to glucocorticoids [61]. In this regard, Feng et al. demonstrated that radiomics may help predict glucocorticoid-sensitive patients. Specifically, the authors reported that the treatment response was 68.5% using the radiomics approach, compared to 36.4–38.5% without radiomics.

**Table 1:** Radiomics in fibrotic interstitial lung diseases: main studies.

Authors/Ref.	Population	Aims	Main Results
Chung JH et al., 2024 [62]	UIP *n* = 2907 pts	To develop a deep learning-based radiomic classifier for usual interstitial pneumonia	1. The DL-based UIP classifier predicted visual UIP in the performance cohort with a sensitivity and specificity of 93% and 86%, respectively; 2. The DL-based UIP classifier predicted visual UIP in the multi-centre ILD clinical cohort with 81% and 77%, respectively.
Refaee T et al., 2022 [49]	ILDs *n* = 328 pts	To evaluate the use of radiomics to differentiate between normal lung tissue and ILDs; To evaluate the use of radiomics to distinguish IPF with a typical or less typical (biopsy-proven) UIP pattern.	Classification between normal, IPF/UIP, and other ILDs using radiomics could help discriminate between different types of ILDs via HRCT, which are hardly recognizable with visual assessments.
Haga et al., 2024 [51]	Fibrotic ILDs *n* = 100 pts	Identify CT radiomics features associated with cellular infiltration; Construct CT radiomics models predictive of cellular infiltration.	The model provided useful information regarding cellular infiltration in LD with a good correlation between SLB specimens (root–mean–square error: 0.797).
Sun H et al., 2024 [50]	ILDs *n* = 279 pts	Applying a 3D lung texture-based structural analysis to help identify f-ILD.	The diagnostic performance of the model was significantly superior to that of the radiologist, both at the CT sequence level and patient level (*p* < 0.05).
Lauer et al., 2024 [63]	Murine models of Bleomycin-induce PF PF-ILDs *n* = 19 pts	To evaluate whether an integrative analysis of delta radioproteomics can be used to stratify the degree of molecular response to nintedanib.	Unsupervised clustering of delta radiomic profiles revealed 2 distinct imaging phenotypes; Integrative analysis of delta radiomics and proteomics demonstrated that these phenotypes reflected different treatment response states; Delta radiomics stratifies nintedanib-treated patients with PF-ILD according to lung function decline.
Qiu J et al., 2024 [64]	Pulmonary Sarcoidosis *n* = 126 pts	To develop a multi-channel, CT, and radiomics-guided ensemble network (RadCT-CNNViT) with visual explainability for pulmonary sarcoidosis vs. LC classification using chest CT images.	The model achieved the highest performance in accuracy, sensitivity, specificity, precision, and F1-score, with combined AUCs of 0.93 ± 0.04, 0.94 ± 0.04, 0.93 ± 0.08, 0.95 ± 0.05, 0.94 ± 0.04, and 0.97, respectively, in a five-fold cross-validation study with pulmonary sarcoidosis (*n* = 126) and LCa (*n* = 93) cases.

Abbreviations: AUC: Area under the curve; CT: computed tomography: DL: Deep Learning; f-ILD: fibrotic-ILD; ILD: Interstitial lung disease; UIP: Usual Interstitial Pneumonia.

**Table 2:** Main studies on radiomic applications in Interstitial Lung Diseases related to Connective Tissue Diseases (CTD-ILDs).

Authors/Ref.	Population	Aims	Main Results
Jiang X et al., 2023 [65]	CTD-ILD *n* = 184 pts	To analyse the feasibility of predicting the GAP stage by radiomics based on chest CT.	The nomogram model that combined clinical factors and radiomics features achieved higher accuracy in both training (88.4% vs. 82.1%) and testing (83.3% vs. 79.2%).
Qin S et al., 2023 [53]	CTD-ILD *n* = 245 pts 2 datasets	To develop a radiomics nomogram for clinical management using the ILD-GAP index system.	The CT-based radiomics nomogram showed favorable efficacy in predicting individual ILD-GAP stages. External validation cohort: AUC, 0.85 (95% CI: 0.720–0.919)
Qin S et al., 2023 [52]	CTD-ILD *n* = 215 pts	To develop and validate a nomogram using clinical features and CT-based radiomic features to predict OS.	Patients with higher radiomic scores had higher mortality risk than those with lower radiomic scores (HR, 12.396; 95% CI, 3.364–45.680; *p* < 0.001); The combined nomogram showed better predictive capability than the clinical model with higher C-indices, time-AUCs, and overall net-benefit.
Feng DY et al., 2018 [66]	CTD-ILD	To identify glucocorticoid-sensitive patients using a radiomics approach.	Radiomics-based predictive models reliably identified glucocorticoid-sensitive CTD-ILD patients.
SSc-ILD			
Joye et al., 2024 [54]	SSc-ILD	Evaluate the efficacy of radiomics-derived srCT scans versus fcCT for diagnosing and staging of ILD SSc; Reduce radiation exposure.	Radiomics from srCT is an effective and preferable alternative to fcCT for Diagnosis: AUC = 0.85 ± 0.08 with srCT, and AUC = 0.83 ± 0.06 fcCT; Staging: AUC = 0.82 ± 0.08 with srCT and AUC = 0.76 ± 0.08 with fcCT.
Bruni C et al., 2024 [67]	SSc-ILD *n* = 79 pts	To quantify lung vascular and parenchymal changes by CT imaging and correlate them with interstitial lung disease (ILD) features.	CT vessel parameters increase in parallel with ILD extent and functional impairment; CT vessel parameters may represent a biomarker of SSc-ILD severity.
Schniering J et al., 2022 [68]	SSc-ILD *n* = 156 pts	To explore CT-based radiomics for disease characterization, risk stratification, and relaying information on lung pathophysiology.	Radiomics-based risk stratification using routine CT images provides complementary phenotypic, clinical, and prognostic information that can significantly impact clinical decision making in SSc-ILD. A high binary qRISSc score, which identifies patients at risk of progression, was reverse translatable from human to experimental ILD and correlated with fibrotic pathway activation.
Martini K et al., 2021 [69]	SSc-ILD	To retrospectively evaluate if texture-based radiomic features can detect ILD; To distinguish between the different disease stages compared to mere visual HRCT.	The correlation between radiomics and the GAP stage, but not with the visually defined features of ILD-HRCT; The combination of two specific radiomic features in a multivariable model resulted in the lowest AIC of 10.73 with an AUC of 0.96, 84% sensitivity, and 99% specificity.
RA-ILD			
Han et al., 2024 [57]	RA-ILD *n* = 177 pts	To construct a radiomics nomogram based on CT using the ILD-GAP index system for clinical management.	1. The model-based CT performed well in distinguishing different ILD-GAP stages (AUC = 0.901 in validation cohort); 2. The nomogram model, combining the radiomics model and serum KL-6, further enhanced the prediction efficiency of GAP staging (AUC = 0.948, training cohort and AUC = 0.923, validation cohort).
Venerito V et al., 2024 [58]	RA-ILD *n* = 30 pts.	To investigate whether features from whole lung radiomic analysis of HRCT can alone predict mortality.	Radiomic analysis can significantly predict RA-ILD patients’ mortality.
*Anti-MDA5 + DM-ILD*			
He W et al., 2024 [59]	anti-MDA5 + DM-ILD *n* = 189 pts (2 intitutions)	To assess the effectiveness of HRCT-based radiomics in predicting 1. RP-ILD; 2. Mortality.	1. Patients were classified into low- and high-risk groups at 50:50 based on the radiomics nomogram; 2. High-risk group patients demonstrated a significantly higher risk of mortality than low-risk group patients in the institution 1 and institution 2 cohorts [HR = 4.117, HR = 7.515, respectively).
Li Y et al., 2024 [70]	anti-MDA5^+^ DM-ILD *n* = 103 pts	To establish a model for the prediction and early diagnosis of anti-MDA5^+^ DM-associated RP-ILD based on clinical manifestations and imaging features.	The combination model built with clinical and radiomic features could reliably predict the occurrence of RP-ILD in MDA5^+^ DM patients. Training set: AUC, sensitivity, specificity, and accuracy: 0.994, 0.966, 0.977, and 0.931, respectively; Testing set: AUC, sensitivity, specificity, and accuracy: 0.890, 0.812, 1.000, and 0.839 in the testing set, respectively.
Xu W et al., 2021 [71]	anti-MDA5 + DM-ILD *n* = 184 pts	To quantitatively assess the pulmonary HRCT images using the radiomics approach; Establish a multidimensional risk prediction model for 6-month mortality.	The RAD score was significantly associated with the 6-month mortality and outperformed the traditional visual score and ILD-GAP score (C-index value, external validation cohort: 0.84 (95% CI, 0.64–1.0).

Abbreviations: Anti-MDA5 + DM-ILD: MDA5-positive dermatomyositis-related interstitial lung disease; AUC: Area under the curve; CT: computed tomography; CTD-ILD: connective tissue disease-associated interstitial lung disease; GAP: gender, age, and pulmonary physiology; fcCT: full-chest CT; f-ILD: fibrotic-ILD; ILD: Interstitial lung disease; KL-6: Krebs von den Lungen; LC: lung cancer; OS: Overall Survival; PF: pulmonary fibrosis; qRISSc: progressive interstitial lung disease; quantitative radiomic risk score; RAD-Score: Radiomic Score; RA: Rheumatoid Arthritis; RP-ILD: predicting rapidly; SLB: Surgical Lung Biopsy; srCT: slice-reduced CT; SSc: Systemic Sclerosis.

## 7. Conclusions

There is no doubt that the application of AI in ILD management is a game changer for both clinicians and patients. By leveraging quantitative image analysis techniques, radiomics has the potential to revolutionise ILD management, ultimately leading to improved patient care and outcomes. By combining clinical data with molecular and genomic information, machine learning models have proven to be valuable tools for early detection, classification, monitoring, and predicting outcomes and treatment responses in ILDs. Incorporating AI into clinical practice is now the challenge. In this respect, multidisciplinary teams remain central to a comprehensive assessment of patients, where ML-based data should be combined with clinicians’ interpretations of patient history, clinical and functional features, and other diagnostic tools.

## Data Availability

Not applicable.
